# Authorship attribution of source code by using back propagation neural network based on particle swarm optimization

**DOI:** 10.1371/journal.pone.0187204

**Published:** 2017-11-02

**Authors:** Xinyu Yang, Guoai Xu, Qi Li, Yanhui Guo, Miao Zhang

**Affiliations:** National Engineering Lab for Mobile Network Technologies, Beijing University of Posts and Telecommunications, Beijing, China; Southwest University, CHINA

## Abstract

Authorship attribution is to identify the most likely author of a given sample among a set of candidate known authors. It can be not only applied to discover the original author of plain text, such as novels, blogs, emails, posts etc., but also used to identify source code programmers. Authorship attribution of source code is required in diverse applications, ranging from malicious code tracking to solving authorship dispute or software plagiarism detection. This paper aims to propose a new method to identify the programmer of Java source code samples with a higher accuracy. To this end, it first introduces back propagation (BP) neural network based on particle swarm optimization (PSO) into authorship attribution of source code. It begins by computing a set of defined feature metrics, including lexical and layout metrics, structure and syntax metrics, totally 19 dimensions. Then these metrics are input to neural network for supervised learning, the weights of which are output by PSO and BP hybrid algorithm. The effectiveness of the proposed method is evaluated on a collected dataset with 3,022 Java files belong to 40 authors. Experiment results show that the proposed method achieves 91.060% accuracy. And a comparison with previous work on authorship attribution of source code for Java language illustrates that this proposed method outperforms others overall, also with an acceptable overhead.

## Introduction

Nowadays with the rapid growth and popularity of Internet, software plagiarism is becoming more and more common. In this context code attribution may be helpful. [1] Authorship attribution of source code is to identify the author of a given source code among a set of candidate known authors. Apart from software plagiarism, it also has practical value in solving authorship dispute, software forensics, and malicious code tracking etc. [2–5]

Source code can be treated as function text to some extent. The expression of source code is less free than text due to complication limitations. However, programmers still leave fingerprints in their source code. [6] For example, if a programmer wrote a sort code once, he would probably use this encapsulated code fragment again when confronted with the same problem. This makes his programming style consistent and also becomes the main reason why programmers can be identified from stylistics analyses. [7]

Authorship attribution has gained wide attention since Krsul’s initial work [8]. To solve this problem, large amounts of source codes belong to candidate authors are dealt for stylistic features to determine the likelihood with the sample to be tested. While this problem has already been studied previously, our work focuses on authorship attribution for Java source code, aiming at achieving higher recognition accuracy with fewer features as much as possible.

In this paper, a novel authorship attribution model is designed and implemented. First of all, feature metrics are defined on the lexical, layout, structure and syntax aspects. The feature space should cover all the aspects of program writing style and its dimensionality also needs to avoid bringing computational complexity. After that, this paper attempts to first introduce back propagation (BP) neural network based on particle swarm optimization (PSO), PSOBP (BP based on PSO) in short, into authorship attribution. Finally, a series of experiments are conducted to evaluate the model effectiveness, with 91.060% accuracy. Moreover, the accuracy, overhead and parameter sensitivity of the proposed method are analyzed in detail.

In summary, the contribution of this paper is the following ones:

- A complete framework of source code authorship attribution based on PSOBP has been proposed, including two main procedures feature extraction and sample classification.
- The extracted features contain not only lexical and layout level metrics, but also structure and syntax level metrics, all scalable.
- A prototype system of the proposed approach and evaluation experiments based on a real-world dataset have been performed, owning a competitive advantage over previous work.

The remainder of this paper is organized as follows. The related work is described in Section 2. Section 3 specifically introduces the source code authorship attribution method using PSOBP. And experimental results are showed and analyzed in Section 4. Finally, we discuss conclusions and future work further in Section 5.

## Related work

At present research in authorship attribution of source code for C/C++ is relatively mature [9], but less systematic work for Java language. In 2004, Ding and Samadzadeh [10] adapted Krsul’s C metrics for Java, that is, programming layout, style and structure metrics, and used statistical process to measure their contribution. The results show that 48 metrics out of 56 extracted metrics are identified as being contributive. However, the authors did not provide the final subset or rank all features. Shortly afterwards Lange and Mancoridis [11] indicated that Ding used mostly scalar metrics derived from source codes, so they formulated their 18 metrics as histogram distributions, with approximately making up one third of Ding’s metrics. But some metrics are somewhat unbounded, for example the indentation categories [12]. Then Shevertalov *et al*. [13] only selected four of Lange’s metrics, leading spaces, leading tabs, line length, words per line, and used genetic algorithm to discretize metrics. The evaluation was carried out with 20 open source developers and over 750,000 lines of Java source codes. But this feature set is also non-reproducible as they did not provide details on the final set. Apart from above papers, there is much valuable work for source code authorship attribution.[14–20] It is worth mentioning that Burrows *et al*. [12] summarized previous classification techniques, either information retrieval ranking or machine learning in 2012, concluding that they obtain around 90% and 85% accuracy respectively for a one-in-ten classification problem. To data for Java source code authorship attribution, the highest accuracy in the related work is achieved by Frantzeskou *et al*. [21] They used 1,500 7-grams to reach 96.9% accuracy classifying 30 programmers. They demonstrated that comments, layout features and naming patterns have a strong influence on the classification accuracy.

The extracted metrics for C/C++ language can also be introduced into the authorship attribution for Java language partially. For example, Aylin *et al*. [9, 22] investigated machine learning methods to de-anonymize authors of C/C++ both on the source code level and the binary code level. They not only made use of lexical and layout metrics, but also took syntactic metrics into consideration. They have already achieved 94% and 98% accuracy with 1,600 and 250 class authors respectively. Recently Wilco *et al*. [6] also proposed to extract structural features from the abstract syntax tree (AST) to identify JavaScript programmers. The accuracy achieves 85% for 34 authors.

## Proposed method

**Fig 1:**
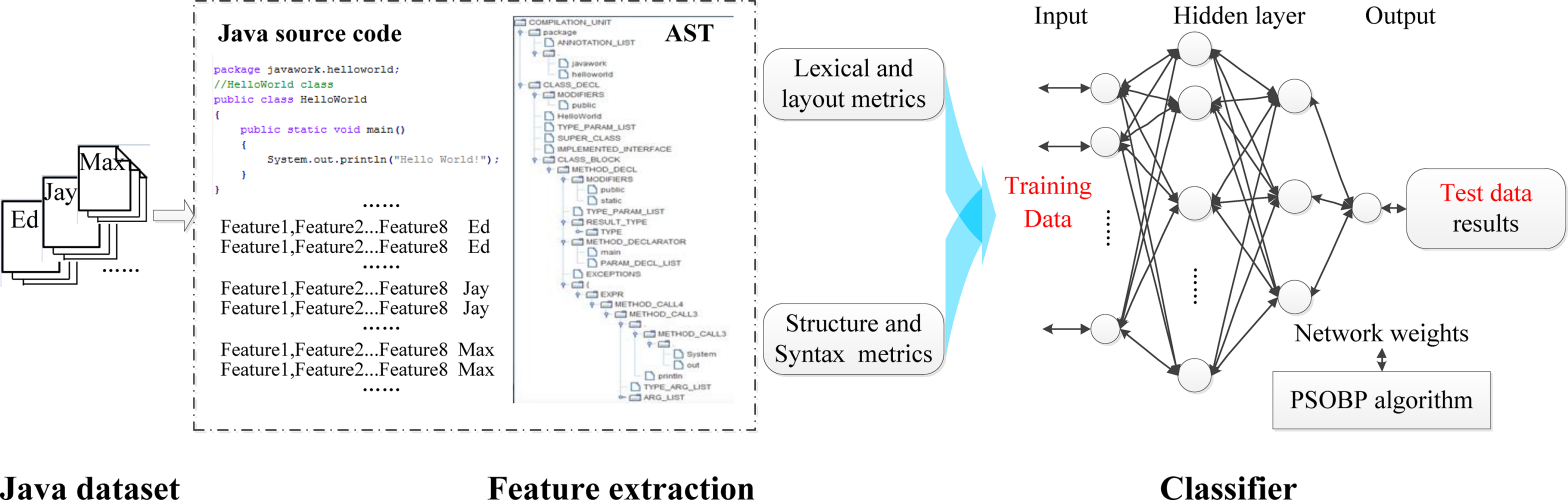
Framework overview.

The goal of source code authorship attribution is to ascribe a specified source code sample to one of candidate authors. Machine learning methods are always used to tackle classification problem. And it is impossible to obtain satisfactory results without appropriate features. To this end, the flowchart of our proposed methods is divided into two procedures shown in Fig 1, namely extracting stylistics features and classifying samples using PSOBP neural network.

First all the collected source codes are pretreated to extract feature metrics one by one Java file. It is necessary that these selected feature metrics should be quite specific to certain programming style, making up a programmer’s problem-solving vocabulary. And at the same time, the features should cover all the aspects of programing style. Feature metrics on lexical, layout, structure and syntax levels are defined. These extracted features form a feature line, representing the Java source file belong to its corresponding author.

Afterwards these processed feature lines are separated into training data and test data on a pro-rata basis, all with author labels. Neural network uses training data to build a classification model, whose network weights are output by PSOBP. It is mentioned that different initial parameter settings have different models, which are adjusted on the basis of engineering experience. Once obtained the expected model, identification results are given using test data. A detailed technical description of all the main steps is given in the corresponding sections.

### Feature extraction

Software programs are analogous to text to some degree, therefore it is quite possible to measure an author’s programming style on the lexical level. But different from pure text it still has structure features. To date, the extracted software metrics used for authorship attribution differ in thousands of ways. Referring to previous work, this paper summarizes feature metrics into two categories, namely programming lexical and layout metrics, structure and syntax metrics. Here, lexical and layout metrics are mainly derived from Ding’s paper [10]. We analyze these metrics combining engineering experience, delete some useless metrics and merge some related metrics into an independent one. For example, “a list of metrics indicating indentation style” (labeled as STY1 in Ding’s paper) includes “percentage of open braces that are along a line” (labeled as STY1a in Ding’s paper), “percentage of open braces that are the first character in a line” (labeled as STY1b in Ding’s paper), “percentage of open braces that are the last character in a line” (labeled as STY1c in Ding’s paper) three petit metrics. But in our paper, these are summarized to a metric “percentage of open braces alone in a line”. It means to compute the percentage of open braces alone in a line to all lines with open braces. This metric can represent how an author expresses nested structure codes. Meanwhile, some metrics are unbounded, for example “average indentation in white spaces after open braces” (labeled as STY1g in Ding’s paper). Some compilers have their own rules therefore making the programming indentation style less free. In this situation, this feature contributes little to identifying a certain author. Thus we decide to delete these metrics. In this paper we define 8 metrics on the lexical and layout level, labeled as PRO and STY in the following Table 1.

**Table 1:** Programming metrics extracted from Java source code files.

Metrics	Description
*PRO1*	Ratio of blank lines to code lines
*PRO2*	Ratio of comment lines to code lines
*PRO3*	Percentage of block comments to all comment lines
*PRO4*	Percentage of open braces ({) alone in a line
*PRO5*	Percentage of close braces (}) alone in a line
*STY1*	Percentage of variable naming without uppercase letters
*STY2*	Percentage of variable naming starting with lowercase letters
*STY3*	Average variable name length
*PSM1*	Ratio of macro variables
*PSM2*	Percentage of “for” statements to all loop statements
*PSM3*	Preference for cyclic variables
*PSM4*	Percentage of “if” statements to all conditional statements
*PSM5*	Ratio of branch statements
*PSM6*	Average number of methods per class
*PSM7*	Ratio of “try” structure
*PSM8*	Percentage of “catch” statements when dealing with exceptions
*PSM9*	Average number of interfaces per class
*PSM10*	Average character number per Java file
*PSM11*	Maximum depth of an AST

Only lexical and layout metrics are not enough to describe the programming style of a single source file comprehensively. The structure and syntax metrics do not confine to text features any longer, but analyze the source code as a whole [23]. The metric extraction relies on abstract syntax tree (AST), which is a tree structure of source code abstract syntax. We totally define 11 metrics on the structure and syntax level, labeled as PSM in Table 1. Finally, after adjusting the metrics according to the classification effect multiple times, 19 metrics are defined totally. An overview of these metrics is given in Table 1. These 19 metrics are either percent or numerical value, all quantitatively scalable. In addition, our software metrics are suitable for both compiled files and source code fragments. But syntax error is not discussed further in this paper.

### Classifier

There is a lot of research work on how to optimize BP neural network to accelerate convergence and avoid local minimum. Particle swarm optimization is one of the optimization algorithms based on swarm intelligence. It shares individual information to make the swarm move towards the optimal solution. In this section, some related knowledge about BP and PSO algorithm is reviewed respectively, to help understand the subsequent method.

#### BP algorithm

BP neural network is currently one of the most widely used neural network models. [24–25] It is a multi-layer feed-forward network trained by the error back propagation algorithm. This means that BP neural network uses the gradient descent method, adjusts the weights and thresholds of the network through back propagation in order to make the quadratic sum of the network error minimum. BP network can learn and store a lot of input-output model mapping, without revealing the mathematical equations of the mapping relationship in advance. In general, BP neural network structure includes input layer, hidden layer and output layer as shown in Fig 1. BP neural network has a strong nonlinear mapping ability, especially suitable for classification or approximation problem.

#### PSO algorithm

BP neural network has a strong self-learning and generalization ability, and also easy to implement, making it often being applied to classification problem. However, BP neural network has several drawbacks, such as slow convergence speed, low prediction ability and locally optimal solution [26]. PSO [27–30] overcomes these above defects and at the same time controls the training time of neural network in a reasonable range. Thus, substituting PSO for gradient descent method to train BP parameters can improve performance greatly. In PSO algorithm, the solution for optimization problem can be treated as searching for the proper “particle”. The procedure is described below. Firstly, the initial solution is generated, i.e. initialize *N* particles in the *D* dimension feasible solution to constitute population *x* = {*x*_1_, *x*_2_,…, *x*_*N*_}. Each particle has two vectors, namely position and velocity, denoted as *x*_*i*_ = {*x*_*i*1_, *x*_*i*2_, …, *x*_*iD*_} and *v*_*i*_ = {*v*_*i*1_, *v*_*i*2_,…, *v*_*iD*_}. Secondly, calculate the fitness value of these particles according to the objective function. In the iteration process, the particle updates two extremes timely, one is *p*_*id*_ the best solution searched by the particle itself, and the other is *g*_*id*_ the optimal solution searched by the population currently. Finally, loop above steps until a satisfied fitness is met or the maximum number of iterations is reached.

The original formulae used for updating velocity and position are shown below in Eq (1) and Eq (2):

$$v_{id}(t+1)=v_{id}(t)+c_{1}\times rand()\times [p_{id}(t)-x_{id}(t)]+c_{2}\times rand()\times [p_{gd}(t)-x_{id}(t)]$$ (1)

$$x_{id}(t+1)=x_{id}(t)+v_{id}(t+1)\;1\leq i\leq n,\;1\leq d\leq D$$ (2)

Where, *v*_*id*_ (*t*+1) represents the *d* dimension velocity of the *i*th particle in generation iteration *t*+1, *v*_*id*_ (*t*) and *x*_*id*_ (*t*) are the *d* dimension velocity and position of the *i*th particle in generation iteration *t*, *c*_1_ and *c*_2_ are the acceleration towards *p*_*id*_ and *g*_*id*_, *r*_1_ and *r*_2_ are the random number between 0 and 1. In order to control the development and exploration ability of PSO algorithm, inertia weight is introduced into Eq (1), forming the standard PSO algorithm as Eq (3). [31]

$$v_{id}(t+1)=wv_{id}(t)+c_{1}\times rand()\times [p_{id}(t)-x_{id}(t)]+c_{2}\times rand()\times [p_{gd}(t)-x_{id}(t)]$$ (3)

It can be seen that *w* controls the influence of previous speed on current one. Large inertia weight makes particles have great speed, owing a strong exploration ability, while small inertia weight makes particles have a strong development ability. To balance the exploration and development ability, inertia weight must be chosen reasonably. In this paper, we leverage the time-varying inertia weight as Eq (4).

$$w=w_{\max}-\left(w_{\max}-w_{\min}\right)/iter_{\max}\times iter$$ (4)

The inertia weight is valued in linear decreasing way. In this formula, *iter* denotes the current number of iterations, *iter*_*max*_ means the largest number of iterations, *w*_*max*_ is the initial value of inertia weight, and *w*_*min*_ is the final value.

#### Enhancing BP with PSO

**Fig 2:**
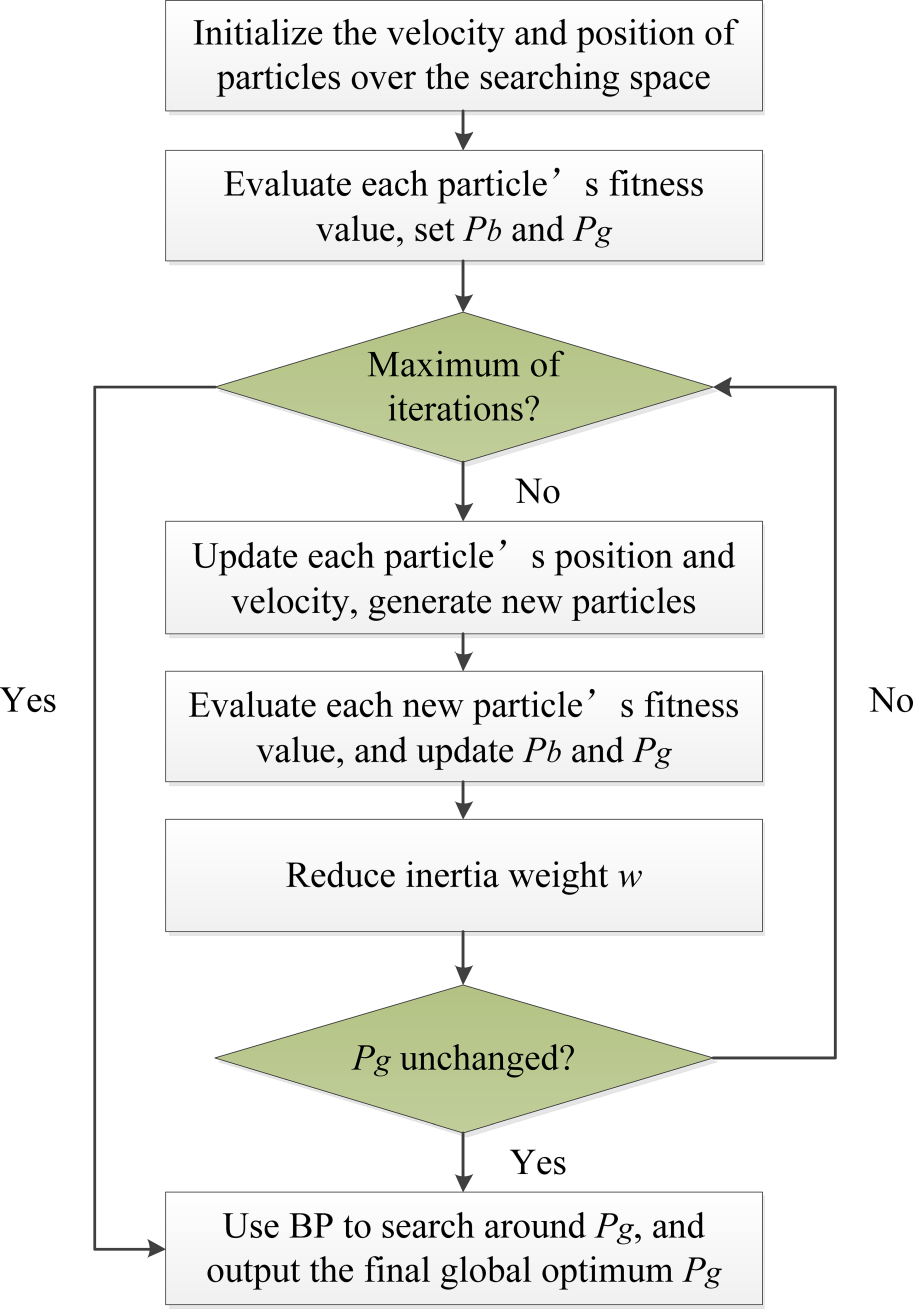
The flowchart of PSOBP.

PSO algorithm has a strong ability to find a global optimal solution. However, the search progress will become slow and even all the particles fall into a local optimal value near the global optimal value, whereas, BP algorithm has the advantage of local searching ability. Thus, PSO and BP algorithm can be combined to make full use of the PSO global search feature and BP local search feature to form a hybrid algorithm PSOBP. In this paper, the searching process of PSOBP is as follows: Firstly initialize a group of particles. Secondly the velocity and position of all the particles are updated according to equations, and a new set of particles are generated. Thirdly these particles are used to search the global best position using PSO algorithm. Finally, BP algorithm is made use of to search around the above global optimum. The flowchart of PSOBP algorithm is also illustrated in Fig 2. In this way, PSOBP algorithm is able to find the optimal solution quickly and accurately.

The PSOBP procedure is summarized as follows:

Step 1: initialize a group of particles randomly over the searching space.
Step 2: evaluate each particle’s fitness value, *P*_*b*_ is set as the position of current particle and *P*_*g*_ is the best position of all the particles.
Step 3: if the maximum of iterations is reached, then go to Step 8, otherwise go to Step 4.
Step 4: store the best position of each particle and global best position, and then update the position and velocity of all the particles according to Eq (2) and Eq (3), thus a new set of particles are generated. If a particle flies beyond the position boundary, then its position will be set *X*_*min*_ or *X*_*max*_; if a particle velocity is beyond the velocity boundary, then its velocity will be set *V*_*min*_ or *V*_*max*_.
Step 5: evaluate each new particle’s fitness value. If the new position of the *i*th particle is better than *P*_*ib*_, then substitute *P*_*ib*_ with the new position, otherwise *P*_*ib*_ stays unchanged. Likewise, if the best position of all new particles is better than *P*_*g*_, the new position is set as *P*_*g*_, otherwise *P*_*g*_ stay unchanged.
Step 6: reduce the inertia weight *w* according to Eq (4).
Step 7: if the global optimum *P*_*g*_ remains unchanged for ten generations, then go to Step 8, otherwise go to Step 3.
Step 8: Use the BP algorithm to search around *P*_*g*_. If the BP search result is better than *P*_*g*_, use the new search result as the final optimum; or else output *P*_*g*_ as the global optimum.

Although PSOBP overcomes the limitations of BP and PSO algorithms, it is still inevitable to exist some drawbacks. Like other optimization algorithms, it has several parameters needing to be adjusted. However, parameter selection is lacking of systematic, standardized theoretical work. In this paper, we set parameter values on the basis of previous work and engineering experience. Fortunately, there are not too many parameters for PSOBP algorithm. In addition, as the problem scale goes larger sharply, the complexity of neural network will increase. At the same time, the classification accuracy will decrease and more running time will be spent. However in this paper, the authors we need to deal with are still small-scale but satisfy practical needs, this phenomenon is not that obvious.

## Experiment evaluation

In the evaluation section, experimental results are present. The authorship dataset section gives an overview of the data we collected. Then we demonstrate the training procedure, including how to use the training data and test data, how to adjust BP and PSO parameters. Afterwards we compare PSOBP and BP, and also evaluate the effectiveness of PSOBP against previous work. Finally, we conclude the evaluation with summarizing the method and providing software engineering insights.

### Authorship dataset

Obtaining a representative dataset for authorship attribution is rather important, thus how to select an appropriate dataset will be a key issue. Our goal is to solve practical author identification problem, so the selected dataset should be close to “ground truth”, and provide sufficient information as much as possible. Unfortunately, there is no such existing dataset for source code authorship attribution. To this end, source code samples are crawled from an open source code website.

Github (accessible at https://github.com//) has become the largest code storage site and open source community in the world, with more than nine million registered users and 21.1 million code repositories. Moreover, whether a repository is committed by a single author or multiple authors is also marked. Here we do not consider the situation where a single source code is completed by multiple programmers, which is out of the scope of this paper. Only these repositories that are contributed by a single author are collected. Although we cannot guarantee that single author codes in Github refer to single authors absolutely, as there are various instances where multiple developers work on the code and commit it by a single author, the noise in the dataset code is evitable. By doing so, each repository is able to represent a single developer roughly, and it is possible to distinguish between multiple developers. The collection was completed in September 2016. Generally speaking, Java repositories are much fewer than C/C++ language. We collected source code samples belong to 100 authors meeting the above restriction. These 100 authors have 1 to 3 repositories, and most of them only have one repository.

**Fig 3:**
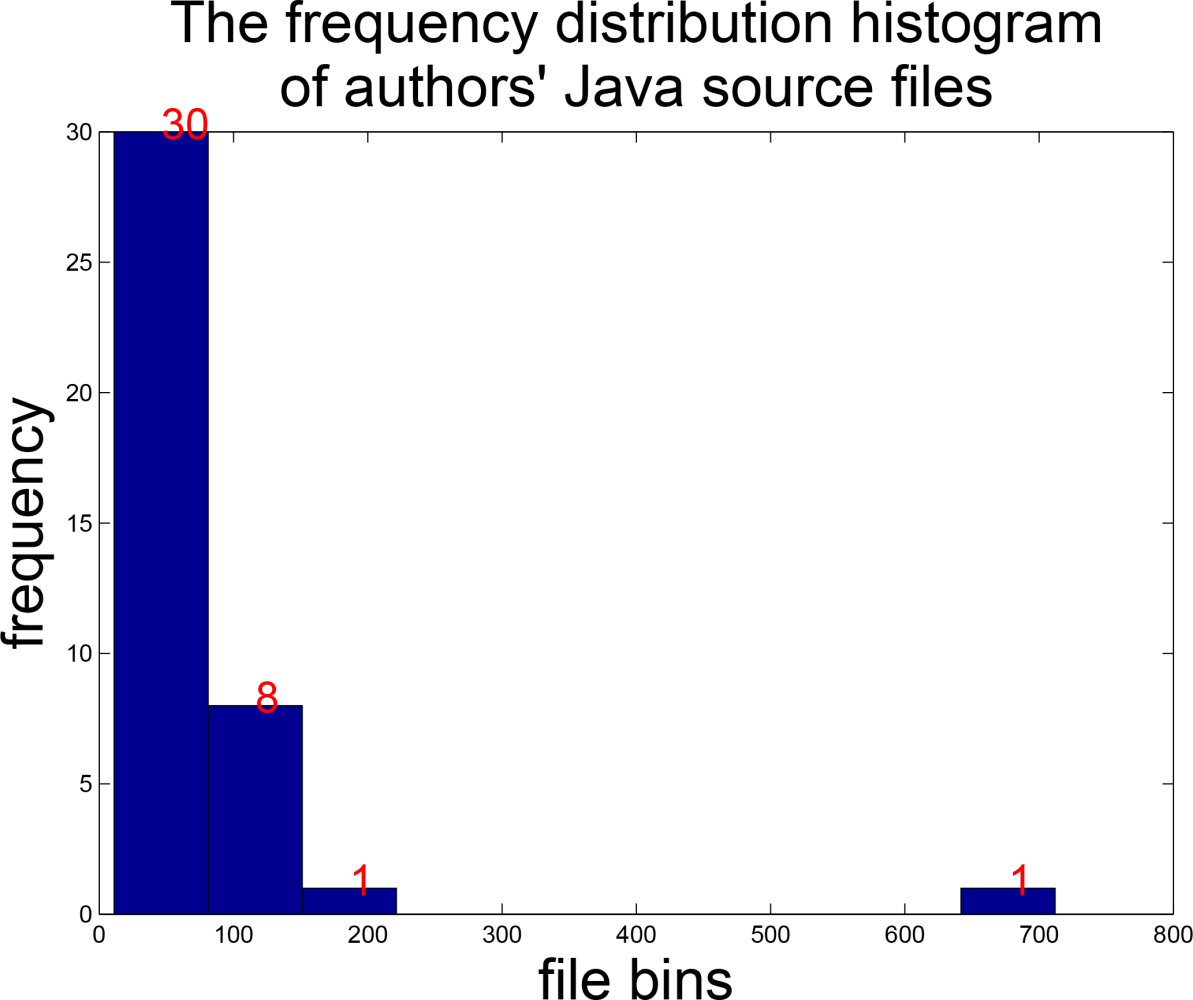
The frequency distribution histogram of Java files.

After collecting the dataset we have carried out data cleaning. On the one hand, some authors only have few Java source code files, bringing difficulty to machine learning. On the other hand, certain parts of Java source code samples are automatically generated by the system, containing no author programming style information. Such data will interfere with the classification accuracy. Therefore, it is essential to take measures to filter the collected dataset. Firstly, we adopt a predefined blacklist of third-party library names, which are crawled from the Maven Repository. Therefore, most of library codes are removed from projects. However, it is hard to find the libraries written by other developers as extension. Secondly, when a programmer develops an Android application to achieve a specific function, he is bound to write his own codes. Although certain parts of the code are generated by the system, for example the abstract class and interface framework code and so on, they account for a small proportion. This noise has little effect on the final classification result. Thirdly, some JUnit test cases are automatically generated while developers will also write their own test cases. In this situation, it is difficult to determine which parts are written by developers themselves. So in this paper, all the JUnit test cases are roughly removed. Finally, the author folders whose total Java source code files are fewer than 10 have also been removed. Through the above data filtering strategies, the authorship dataset comprises 3,022 Java files with 40 authors. For learning and study purposes, we have published the experimental dataset (accessible at https://github.com/buptlearner/authorship_attribution). The minimum file number that an author contributes is 11, and the maximum is 712. The frequency distribution histogram of 40 authors’ Java source files is shown in Fig 3. Three quarters of total authors own less than 81 files, and 8 authors have source code files ranging from 81 to 151. These two intervals make up the vast majority of all the data. There is one author owing 201 files, and only one author has the largest number of files, 712 source codes. This data distribution conforms to actual situation. In addition, statistics data shows that the average line length is 98.63, ranging from 16 lines to 11,418 lines.

### Training procedure

In machine learning the classification model should not only be suitable for training data, but also most importantly able to make reliable predictions on general untrained data, thus it is necessary to avoid over-fitting problem. To this end, several measures are taken. First, in normal conditions the more adequate training data is, the better a classification model is. In the training procedure the authorship dataset has provided enough data points for PSOBP to train a proper classification model. Second, in the model design phase the dimension of input feature vector and hidden neurons are controlled in a reasonable range. Third, in order to make full use of the dataset and eliminate the effect of sample choosing, cross-validation is used to evaluate the effectiveness of the classification model.

In the initial phrase, particles are randomly over the search space, ranging from -1 to 1, and PSO and BP parameters are also set at this time. As PSOBP is sensitive for initialization parameters [32–34], different initialization parameters will obtain different classification models. In this paper, these important parameters of PSO and BP are adjusted using controlling variable method. Table 2 lists their names, corresponding definition, note and value used in this experiment. The population size *N* is usually set between 20 and 40. Experiments show that for most of problems, 30 particles can achieve satisfactory results, but for certain difficult problems, it can also be set 100 or 200. In this paper, population size is 100. The particle length *D* is the size of problem, determined by the specific optimization problem. *D* = (*indim*+1)×*hiddennum* + (*hiddennum*+1)×*outdim*, *indim*, *hiddennum* and *outdim* mean the neuron number of input layer, hidden layer and output layer respectively. The maximum velocity *V*_*max*_ determines the maximum distance that a particle can move in a single iteration. The maximum velocity must be limited, otherwise a particle might run out of the search space. *V*_*max*_ is usually set to the width of the particle range. After adjusting several times, we set *V*_*max*_ 1 and *V*_*min*_ -1 in this paper. The inertia weight *w* decreases as Eq (4), and let the initial *w* be 0.9. The acceleration constants, both *c*_1_ and *c*_2_ are 1.49, different from the default setting 2.0. *r*_1_ and *r*_2_ are two random numbers in the range of [0,1].

**Table 2:** Key parameters of PSO algorithm.

Name	Definition	Note	Value
*N*	Population size	Usually 20~40	100
*D*	Particle length	Determined by the optimization problem	Design formula as above
*V*_*max*_	Maximum velocity	Maximum velocity limit in each dimension	1
*w*	Inertia weight	Linear decreasing weight generally from 1.5 to 0.5	Eq (4) *w*_*max*_ = 0.9, *w*_*min*_ = 0.4
*c*_1_,*c*_2_	Acceleration constant	Usually both 2.0	*c*_1_ = *c*_2_ = 1.49
*r*_1_,*r*_2_	Random number	Between 0 and 1	Random number

The parameters used in this paper are not default configurations, they are adjusted according to the specific authorship attribution problem. Hence, we make use of controlling variable method to compare the result against several parameters configurations. For example, we change the maximum velocity and keep the other parameters the same as our final configuration. Then parameters are determined according to classification results. In order to avoid the influence of accidental factors such as random variables, experiments should be repeated several times. Therefore each time a variable is adjusted we carry out experiments three times and take the average shown in Table 3. For population size, inertia weight, acceleration constants, we also repeat the operation. In the comparison procedure, the training data and test data is 3:1. Table 3 lists the classification result of different parameter configurations. It is worth mentioning that actually for each single variable, we try continuous data, but only a few default settings are listed.

**Table 3:** The effect of different parameter configurations.

Single variable	Classification accuracy
*V*_*max =*_ 10	89.073%
*N*_*=*_ 40	88.571%
*w*_*max*_ = 1.8	88.711%
*c*_1_ = *c*_2_ = 2.0	87.215%
**Our final configuration**	**90.659%**

**Fig 4:**
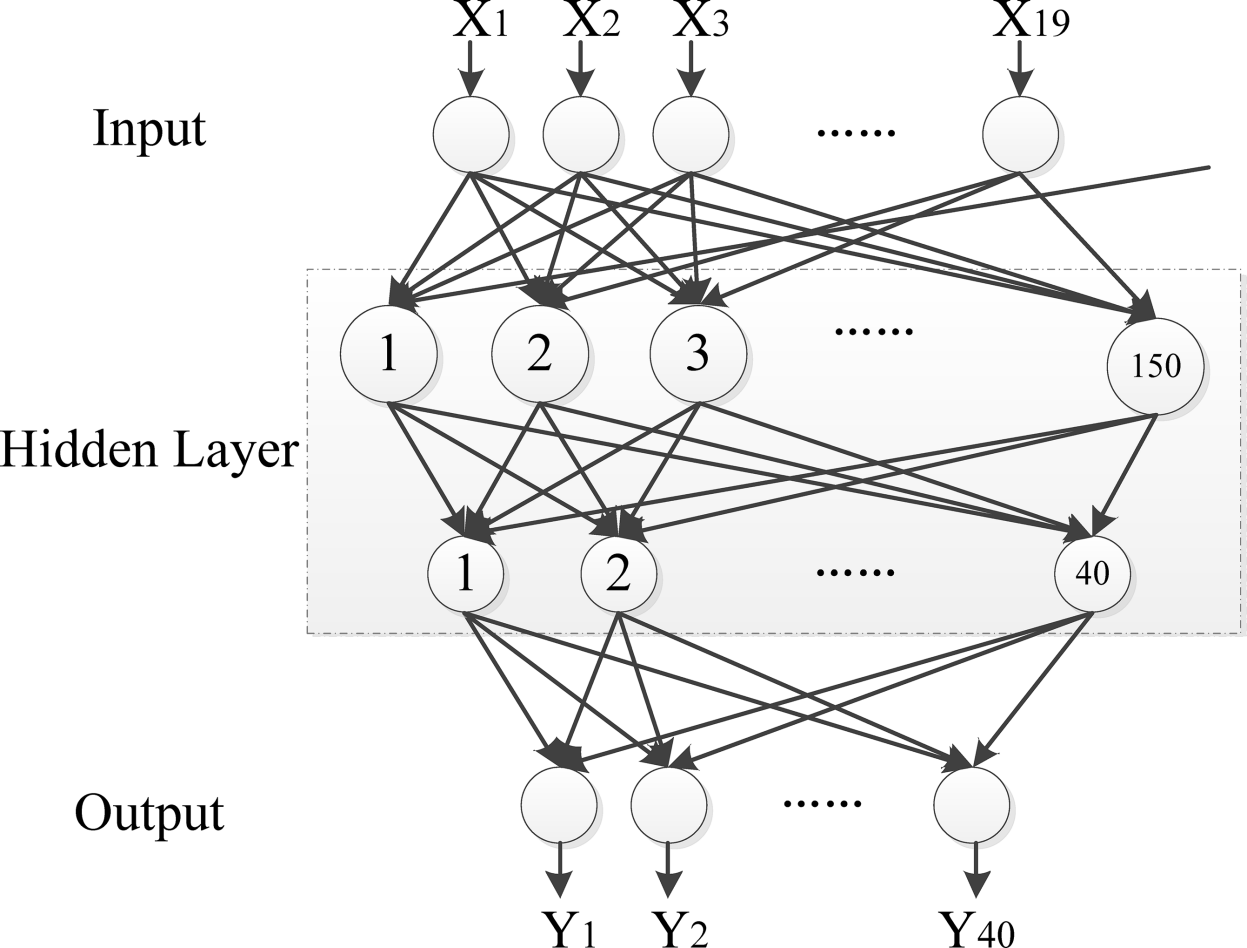
The structure of neural network.

In addition of parameters, the structure of neural network plays an important role in building an appropriate model. The neural network has three layers, input layer, hidden layer and output layer as in Fig 4. The input layer contains 19 neurons, corresponding to 19 dimension features, all numeric value. The hidden layer has 150 neurons. The hidden layer structure is determined according to empirical formula and engineering experience. The output layer contains 40 neurons, corresponding to 40 authors. The function of hidden layer and output layer has a great influence on neural network prediction precision. Generally, the function of hidden layer nodes is *logsig* or *tansig*, and the function of output layer nodes is *tansig* or *purelin*. In this paper, the functions of hidden and output layer are both *tansig*.

### Classification comparison of PSOBP and BP

Cross-validation is employed multiple times to evaluate the effectiveness of our proposed method. In this experiment we take ten 10-fold cross-validation. Specially, we divide the original dataset into 10 subsets. Each time a subset is treated as validation data in turn and the others are reserved for training. All of them are labeled with corresponding author numbers. On this basis, we obtain ten sets of training data and validation data. PSOBP and BP neural networks are used to get classification models using training data, and validation data is used to evaluate the model accuracy. If the predicted output is in accordance with the actual author number, we determine that this sample is classified correctly, otherwise wrongly. We take the average as the accuracy of a 10-fold cross-validation. The results of ten 10-fold cross-validation for BP and PSOBP are illustrated in Table 4 respectively. Due to stochastic nature of the PSOBP algorithm, mean value and standard deviation instead of each accuracy value are given. Taking cross validation can avoid over-fitting effectively, making the result more convincing. Finally, PSOBP achieves 91.060%, higher over BP 76.093%. When the classification accuracy is more than a certain value, it will be not that easy to be improved further. But it performs relatively stable, no significant ups and downs.

**Table 4:** Cross validation accuracy of BP and PSOBP neural network. (percentage %).

Counter	Mean value	Standard deviation	Mean value	Standard deviation
*k* = 1	75.913	2.477	91.218	4.493
*k* = 2	76.246	3.402	91.342	4.060
*k* = 3	75.944	2.940	90.567	6.067
*k* = 4	75.969	4.156	91.001	4.394
*k* = 5	76.050	3.197	91.008	4.682
*k* = 6	75.945	3.027	91.093	6.046
*k* = 7	76.439	4.606	91.106	5.018
*k* = 8	76.507	2.476	91.080	4.444
*k* = 9	75.785	2.056	91.013	5.331
*k* = 10	76.132	3.420	91.172	4.152
	**BP: 76.093**		**PSOBP: 91.060**	

**Fig 5:**
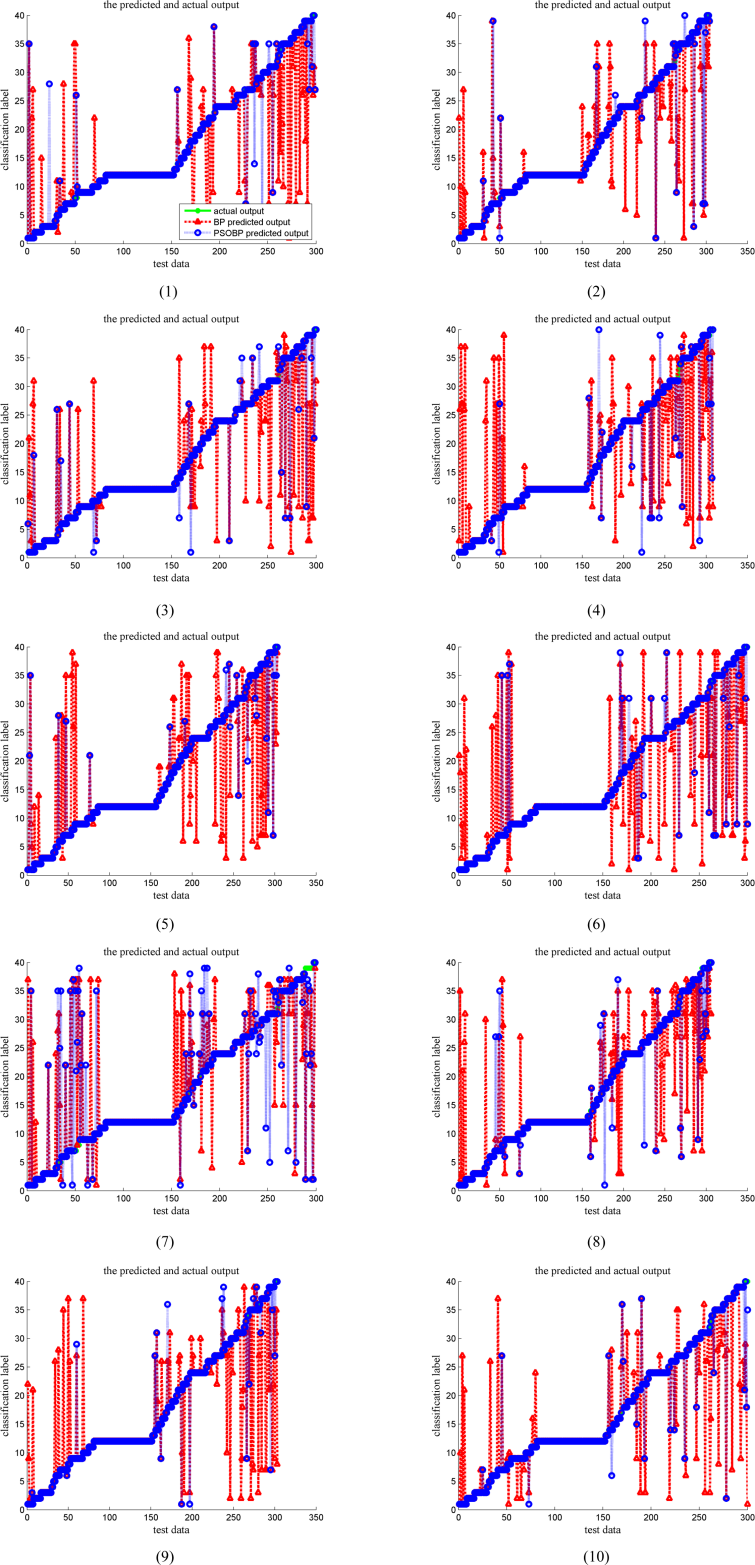
The classification results of PSOBP and BP neural network in one 10-fold cross-validation experiment.

It can be seen that in Fig 5(1–10) the x-axis is just the validation data of one 10-fold cross-validation, one tenth of the total Java source code samples. And the y-axis represents the author number, ranging from 1 to 40. Restricted by the limited space in figures, we only add one legend in Fig 5(1). The legend displays that, the green solid line means the actual output, numerical growth in discretization. The red triangle line stands for the BP prediction output, and the blue circular line represents the PSOBP prediction output. It is obvious that PSOBP outperforms than BP on the same given validation data. The PSOBP predicted output overlap the actual output in the vast majority of cases. It is worth mentioning that in our experiment PSOBP and BP use the same common parameters, including neurons, training epochs, learning function, experiment error and so on.

However, in a 10-fold cross-validation certain experiment accuracy is obviously lower than the others, making standard deviation not that small. Examining its corresponding original author samples, we make the following analyses. The source codes derived from Github may not belong to their claimed authors completely, as some source code fragments may be copied and pasted from existing codes. And some authors may come from a same organization, which may have detailed and strict requirements for programming style. Thus in this situation it is difficult to distinguish two authors from the stylistic features, which make up the main part of our proposed feature space. That is the reason why the accuracy of certain test can be relatively lower. But the overall accuracy is satisfactory and in practice a more accurate result can be obtained combined with artificial analyses.

### Comparison to previous work

There are several classical machine learning approaches often used for classification problem. Conducting a number of comparison experiments to demonstrate the effectiveness of our proposed approach is essential. In this comparison procedure, our collected dataset is split into training data and test data to classify programs from 40 authors. The former account for 75% (2,267 Java source code files) while the latter constitute 25% (755 Java source code files). Repeat the experiment multiple times and take the average as the final result. Both accuracy and running time are considered, listed in Table 5.

**Table 5:** Comparison to other classifiers.

Classifier	Accuracy	Running time (s)
Random Forest	79.735%	9.679
Support Vector Machine	73.642%	201.220 ^a^
Naïve Bayes	49.007%	11.974
BP	75.107%	48.200^a^
**This work**	**90.659%**	**582.812**^**a**^

^a^ Including the time spent in optimization procedure.

It can be seen that with the same other conditions, PSOBP accuracy is higher than others achieving 90.659%, although it takes much more time. The time spent in our work is more than other AI classifiers, but they are all within a reasonable and tolerant range. Further we analyze that these extra time is mostly spent in the process of searching for the global optimal solution, including searching for appropriate network parameters and neural network optimization. Once finding an optimized NN model, it takes only 0.38 seconds on average to judge the authors of given test samples for PSOBP. It is intuitive that with the problem size increases, the particle scale, hidden neurons, and other parameters should be adjusted accordingly. These all lead to time increasing. In this paper, we aim to identify 40 authors, and the time is reasonable for the problem size.

There is a number of related work as summarized in Section 2 in the literature. In this paper, we aim at solving authorship attribution of source code written by Java language. To our best knowledge we summarized the typical and all Java source code authorship attribution work in Table 6. Generally speaking, we significantly outperform them according to the number of classification programmers and corresponding accuracy. However, we notice that Frantzeskou *et al*. identified 30 programmers, achieving 96.9% accuracy, but the average lines of all source code files in their dataset (172 lines of code on average) are longer than ours (98 lines of code on average).With the author scale slightly larger, the accuracy of our proposed method still remains relatively high.

**Table 6:** Comparison to previous work.

Related work	# of Programmers	Results
Ding and Samadzadeh [10]	46	67.2%
Lange and Mancoridis [11]	20	75%
Shevertalov *et al*. [13]	20	75%
Frantzeskou *et al*. [21]	30	96.9%
**This work**	**40**	**91.1%**

At the same time, compared to recently published work for other popular languages, for example C/C++/JavaScript, the experimental results of our propose method are also valuable. In the reference [22], Aylin *et al*. de-anonymized authors of C/C++ achieving 94% and 98% accuracy with 1600 and 250 class authors respectively. However, the collected Java language repositories are much smaller than C/C++, leading to the accuracy decrease of machine learning classifiers correspondingly. It is inevitable unless expanding the dataset. Wilco *et al*. [6] identified JavaScript programmers with 85% accuracy for 34 authors. Taken together, results in this paper can satisfy practical engineering needs.

### Results discussion

In this section, we summarize the conclusions drawn from the above experiments. In particular, the difficulty of this problem, the effectiveness and limitations of our current approach are discussed comprehensively.

#### Problem difficulty

The experiment collecting all authors’ repositories from Github to date resembles a real world scenario. The Java repository is scanned from end to end to ensure that it belongs to a single author. In such an experiment setting, the collected dataset excludes those repositories that are contributed by multiple authors or forked from others’. Thus the limitation of the dataset does not allow us to assess the effect of attributing code samples completed by multiple developers. This is also beyond the scope of our study. However, we are convinced that these defined features will also have a reference value for multiple author classification problem.

There are fewer Java files than C/C++ generally. Furthermore, after the data filtering procedure our final dataset is with fewer authors, fewer average Java files than most datasets used for C/C++ authorship attribution. This brings challenge to the subsequent machine learning classification method. Moreover, there are varieties of programming features for authorship attribution, but not all of them contribute a lot. It should also be considered carefully which subset of features will be chosen.

#### Method effectiveness

Multiple research groups have published source code authorship attribution work so far. Their experiment environment and evaluation methodologies vary greatly, making it difficult to judge which one is the most accurate. But a series of comparison experiments are conducted. Our proposed method performs better than theirs generally, especially appropriate to deal with collections of moderate size. In conclusion, we use relatively fewer features, feasible method to achieve our goal.

#### Parameter sensitivity

No matter PSOBP or BP will be affected by parameters a lot. Given a set of data samples, these parameters should be adjusted according to the problem to be solved. But once the classification model is built, it will no longer change. The parameter tuning of PSO and BP is also studied in various work, in this paper we set these adjustable parameters according to these empirical conclusions and engineering experience. As the parameters used in this paper are not default configurations, we carry out a series of experiments to validate the parameter effectiveness. Meanwhile, we also give an explanation about the meanings of these parameters.

## Conclusion

De-anonymizing programmers has practical meaning when source codes are available. To this end, a new approach based on PSOBP to authorship attribution of source code has been present. First, 19 dimension feature metrics are defined systematically and comprehensively. Not only feature metrics on the lexical and layout level are contained, but also structure and syntax feature metrics are taken into consideration. And these features are language specific, aiming at expressing Java properties.

Then we first introduce back propagation neural network based on particle swarm optimization algorithm to authorship attribution of source code. The proposed method uses neural network to build a classification model, whose weights are output by PSOBP algorithm.

Finally, a prototype system is devised and implemented. At present no suitable existing dataset is available for authorship attribution of source code. Thus our evaluation experiments are carried on a collected dataset crawled from the open source website Github. It comprises 3,022 Java files belong to 40 authors. The average line length of these Java files is 98.63, ranging from 16 lines to 11,418 lines. On this dataset, our proposed method can achieve a higher accuracy 91.060%, overall outperforming previous work for identifying Java programmers. And the spent time is also within a reasonable range.

In summary, our proposed method can assist authorship attribution of source code work. In the future work, we plan to investigate if these proposed feature metrics still contribute to identify authors of executable binaries. Also, other variant algorithms of PSO and new optimization algorithms combined with BP will be studied for better performance in the authorship attribution of source code field.
